# Epidemiologic Study of *Enterobius vermicularis* Infection among Schoolchildren in the Republic of Marshall Islands

**DOI:** 10.1155/2021/6273954

**Published:** 2021-08-02

**Authors:** Chia-Kwung Fan, Pasaikou Sonko, Yueh-Lun Lee, Ai-Wen Yin, Ting-Wu Chuang, Ramson Kios, Ying-Ting Wang, Chia-Mei Chou, Shao-Lun Hsu, Mai-Szu Wu, Jia-Wei Lin, Chia-Ying Tu

**Affiliations:** ^1^Department of Molecular Parasitology and Tropical Diseases, School of Medicine, College of Medicine, Taipei Medical University, No. 250, Wu-Xing Street, Taipei 110, Taiwan; ^2^Graduate Institute of Medical Sciences, College of Medicine, Taipei Medical University, No. 250, Wu-Xing Street, Taipei 110, Taiwan; ^3^International Master/Ph. D. Program in Medicine, College of Medicine, Taipei Medical University, No. 250, Wu-Xing Street, Taipei 110, Taiwan; ^4^Research Center of International Tropical Medicine, College of Medicine, Taipei Medical University, No. 250, Wu-Xing Street, Taipei 110, Taiwan; ^5^Cell Physiology and Molecular Image Research Center, Wan Fang Hospital, Taipei Medical University, No. 111, Section 3, Xinglong Road, Wen-Shan District, Taipei 116, Taiwan; ^6^Department of Microbiology and Immunology, School of Medicine, College of Medicine, Taipei Medical University, No. 250, Wu-Xing Street, Taipei 110, Taiwan; ^7^Department of Public Health, Ministry of Health, PO. Box 3885, Majuro, Mh96960, Marshall Islands; ^8^Taiwan Health Center in Majuro, Ministry of Health, PO. Box 3885, Majuro, Mh96960, Marshall Islands; ^9^Superintendent Office, Taipei Medical University-Shuang-Ho Hospital, No. 291, Zhongzheng Rd., Zhonghe District, New Taipei City 23561, Taiwan; ^10^Department of International Medical Affairs, Taipei Medical University-Shuang-Ho Hospital, No. 291, Zhongzheng Rd., Zhonghe District, New Taipei City 23561, Taiwan

## Abstract

The prevalence and risk factors of *Enterobius vermicularis* (pinworm) infection among primary schoolchildren (PSC) in the Marshall Islands remain unknown; thus, investigation on the status of pinworm infection rate is necessary to establish baseline data. After parents'/guardians' consent, a total of 346 children (179 boys and 167 girls) participated in this study. Individual's perianal area and thumbs were inspected by using the Scotch tape technique and cellophane tape method, respectively. For each child, demographic and risk factor data were collected by a structured questionnaire and statistically analyzed. The overall prevalence of pinworm infection was 12.14% (42/346). Univariate analysis indicated significant differences in PSC who live in an urban area compared to those who live in the rural area (*p*=0.01). Multivariate analysis still found that PSC who live in the rural area had higher chances to acquire pinworm infection. However, no risk factors were identified to be associated with personal hygiene, sibling number, and parent's educational level or occupation. Nevertheless, a pinworm-like egg was detected on the thumb of one male participant. Children living in the rural area and thumb-sucking behavior are two of the important risk factors of transmitting pinworm infection in the PSC in the Marshall Islands. We suggested an urgent and continuous provision of adequate hygienic sensitization in the school and the community.

## 1. Introduction

*Enterobius vermicularis* (pinworm) is an intestinal parasite that may cause enterobiasis, which is common among primary schoolchildren (PSC) in many countries [1, 2]. Human infection was directly associated with the ingestion of infective eggs through oral routes or from contaminated clothes and bed linens. Additionally, transmission through the respiratory tract has also been speculated by inhaling dust contaminated with eggs [3]. The life cycle of pinworm is uncomplicated, utilizing the human host gastrointestinal tract to become adults, and the gravid female worm migrates to the anus to lay fertilized eggs at night. In some cases, the ovulating female pinworm may also move to the external genitalia to produce fertilized eggs [4].

Enterobiasis is considered the most prevalent helminth infection, with an estimated 1000 million cases worldwide [5]. However, compared to most intestinal helminths, the prevalence of pinworm infection is underestimated because of parasite migration during the night and the difficulty of egg detection in routine stool examination. The burden of pinworm infection among PSC worldwide is not well studied. Worldwide epidemiological studies showed the pinworm infection among schoolchildren in Asia, e.g., 54.9% in China [6], 8.8% in Thailand [7], 47.2% in Myanmar [8], and 4.4% in South Korea [9]; Africa, e.g., 26.3% in Tanzania [10], 1.7% in Angola [11], and 3.3% to 11.7% in Nigeria [12]; South America, e.g., 35.2% in Chile [13] and 29.1% in Argentina [14]; and Europe, e.g., 17.4% in Germany [15], 3.4% in Slovakia Republic [16], and 19.3% in Osh Oblast, Kyrgyzstan [17]. The stool examination showed a low prevalence of pinworm infection as the worm's eggs are sticky and adhered to the perianal skin and clothes. A survey compared the prevalence of pinworm infection using fecal analysis (0.4%) to Scotch tape (45.3%) method in the mountainous Qwa-Qwa State, South Africa, among hospitalized children, suggesting that fecal examination underdetermines the true prevalence [18]. In the United States, pinworm infestation is the most common helminth infection, with over 40 million people being infected [19].

In general, the pinworm infestation is mostly asymptomatic, although symptomatic cases are associated with nocturnal anal pruritus; as a result, the patients pick eggs with their fingernails by scratching the anus causing autoinfection; although rare parasites may invade into the reproductive organs and peritoneal cavity [20, 21], hepatic enterobiasis which is characterized by the existence of granulomas in the liver containing adult helminths or eggs [22, 23] and acute appendicitis [24] can be found uncommonly. Ectopic movements have also been reported to be associated with recurrent urinary tract infection, which may cause secondary bacterial infection in the gastrointestinal tract [25]. These symptoms are accompanied by insomnia, restlessness, instability, and sleepiness during the daytime among infected children.

There are effective antihelminth drugs for pinworm infection. The treatment required a dose of mebendazole. Moreover, albendazole or pyrantel pamoate is an effective drug line. The treatments also require treating close proximity families. Increasing personal hygiene such as washing hands before eating or after toilets and cleaning the bedroom, bed, and clothes is preventive control to reduce the risk of recurrence [19].

Previous studies have stated nine different intestinal parasite infections among schoolchildren were found in the Marshall Islands, but the real pinworm infection rate is unclear [26]. Since PSC are the most vulnerable population infected with pinworm, the present study aims to investigate the prevalence and risk factors associated with *E. vermicularis* infection among PSC in the Marshall Islands.

## 2. Methods

### 2.1. Study Population and Sample Collection

This study was conducted from October to November 2018, and the total PSC population was from 14 primary schools in Majuro City, Republic of Marshall Islands (RMI). Majuro City is situated in the central Pacific Ocean (4°; 14° North latitude and 160°; 173° East longitude). The contents of the leaflets, questionnaire, and consent forms were explained to the parents/guardians of each participant before the commencement of pinworm screening. A total of 1000 consent forms were issued, of which 360 were signed, and subsequently, 346 children (179 boys and 167 girls) voluntarily participated in the study. The sample size was determined using the general formula, *n* = *z*^2^*p* (1 − *p*)/*d*^2^, where *n* is the sample size, *z* (1.96) is the standard deviation with a 95% confidence interval (CI), *p* is the prevalence (23%) based on the infection rate of intestinal parasites in the RMI from a previous study [26], and *d* is the allowed relative error (0.05) [27]. The minimum sample size for the calculation was 273.

Before the commencement of the anal collection, two thumbs from each participant PSC were inspected by the cellophane tape method. Thereafter, the Scotch® tape technique was employed to collect anal samples before the child's bottom was washed on the sampling day. The samples were collected by a trained medical technician from Taipei Medical University. Briefly, the Scotch tape technique involves adhesive Scotch tape onto the glass and touch around the anal area to pick up eggs. The contents of the tape are transferred onto a glass slide for pinworm examination using a standard light microscope. Infected children and their families were given a single dose of albendazole (200 mg) and followed up after one week posttreatment.

### 2.2. Risk Factor Survey

Through the assistance of public health nurses, the participant PSC was interviewed with a structured questionnaire concerning demographic data (gender, age, number of siblings, residence, parents' educational level, and occupation) and personal hygiene (washing hands before eating or after using toilets, finger sucking or keeping fingernails short, bathing habits, household cleaning, and living conditions).

### 2.3. Ethics Approval and Consent to Participate

The research protocols were approved by the Institutional Review Board of Shuang Ho Hospital, Taipei Medical University (TMU-JIRB no. N201805032), and they were also approved by the MOH RMI. An informed consent form was obtained from participant parents/guardians to allow their kids to participate in this project.

### 2.4. Statistical Analysis

Differences in variables data were analyzed using SAS v.9.3 statistical software. The demographic data were determined by a chi-square (*x*^2^) test. The factor associations between the independent variables were determined by logistic regression (odds ratio (OR) and 95% CI) and when *p* value < 0.05 was considered statistically significant.

## 3. Results

The demographic characteristics of PSC were examined for pinworm infection as shown in Table 1. Of the 346 children examined, 42 were found positive, and the overall prevalence was 12.14%. Compared to the prevalence (5.93%, 7/118) in children who lived in an urban area, a significant difference was found in those who lived in a rural area (15.35%, 35/228) and were more likely to have pinworm infection (*p* < 0.05). However, there was no significant relevance in the rate of infection by family background and parent's occupation status (*p* > 0.05). Although no significant difference was detected for age and gender, the prevalence of pinworm infection was more common among children aged >8 years (12.32%, 25/203) than those whose age was ≤8 years (11.89%, 17/143) (*p* > 0.05) as well as slightly higher among girls (13.17%, 22/167) than boys (11.17%, 20/179) (*p* > 0.05).

**Figure 1:**
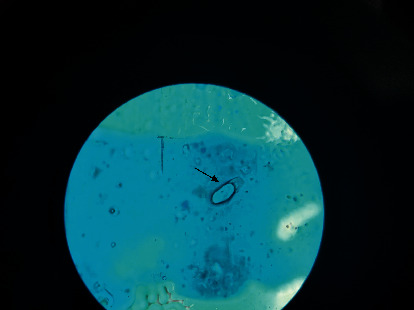
A pinworm-like egg (arrow) was detected from the thumb of a boy.

The univariate characteristics of personal hygiene risk factors were assessed by logistic regression as shown in Table 2. Among the children infected with pinworm, no significant differences were associated with washing hands before eating or after using the toilet, finger sucking, long fingernails, bathing habits, sanitary housing conditions, bed matting, and sharing bed or room with the family member (*p* > 0.05). The multivariate logistic regression analyses of the risk factors associated with pinworm infection among PSC in the Marshall Islands are shown in Table 3. Children who lived in the rural area showed a higher possibility for the acquisition of pinworm infection compared to those who lived in the urban area (OR = 0.35; 95% CI = 0.13–0.93; *p*=0.03). Other factors did not show significant relevance to pinworm infection (*p* > 0.05). However, pinworm-like egg contamination was detected on the thumb of a boy (Figure 1), and he also showed positive in the anal screening.

## 4. Discussion

Pinworm infection is a public health problem in many countries, irrespective of socioeconomic status. The infection is often common among communities like children's care centers (orphanages and kindergartens), schools, and overcrowded households [28–31]. Pinworm infection is considered the most frequent helminth infection in the USA among elementary schoolchildren (6–15 years) attending outpatient clinics [13]. A higher prevalence of pinworm was also reported among schoolchildren (aged 2–12 years) from Gaozhou City, China [6], urban and rural areas of Manila city, and patients in Germany [15].

Intestinal helminths have been reported to be common among PSC in the Marshall Islands, with an overall prevalence of 22.75% [26]. The study indicated six pathogenic helminths, including pinworm. However, only one case has been detected from the stool examination, which seems likely to indicate that the pinworm infection rate is quite low (0.25%, 1/400) [26]. Nevertheless, this is not the real situation as what reflects from the stool's findings because several studies have indicated that eggs are only found in the stool of 5% of infected persons [19]; thus, the Scotch tape test can serve as a quick and sensitive way to clinch a diagnosis.

Compared to other Asian countries, the present study found that the prevalence of pinworm infection in schoolchildren (12.14%) was considerably lower than that conducted in China (>50%) [6], but higher than that in Thailand (7.81%) [32] and Taiwan (0.21%) [31]. Thus, it can be concluded that the pinworm infection is moderately high and, therefore, remains an important parasite disease in Marshallese children. A significant difference was observed in children who lived in the rural area, but not in other variables such as gender, age, and personal and household hygiene. This finding can be explained by that the student number per class in a school located in the rural area is more than that in an urban area; thus, the transmission rate could be high in such a crowded condition [31].

In this study, the risk factor is associated with the area of settlement, but not associated with washing hands before eating or after using the toilet, finger sucking, long fingernails, bathing habits, housing, sanitary condition, bed matting, sharing a bed in a room with the family member, number of siblings, and parents' occupation. In contrast to our results, other reports showed the risk factors of pinworm infection associated with age, hand washing before the meal, bed linens, parents' occupation, and educational level [6, 15, 31, 32]. An interesting finding in the present study is that pinworm-like egg contamination was detected on the thumb of a male participant. A study conducted in South Africa showed the risk factors of pinworm infection among schoolchildren associated with hand contamination [33]. Therefore, the finger-oral route remains an essential indicator of pinworm transmission. The promotion of good personal hygiene and sanitation may decrease the positive infection rate [34, 35].

In Taiwan, the prevalence of pinworm infection among children is considered drastically reduced from 19.9% (1986) to 2.4% (2007) after long-term population-based control [36]. In 2017, the follow-up work stated that the prevalence of pinworm infection among pre-schoolchildren in the 12 districts of Taipei is further reduced to 0.21% [31]. These mass drug deworming and long-term population-based control conducted by Taiwanese can be a strategy to control pinworm infection in Marshallese children. It is noteworthy that the public health sector of the Marshall Islands requires an effective intervention strategic program and prioritizes the parasitic infection among children through community sensitization, routine screening, and active deworming process. The parasitic infection among children not only affects child health and growth but also leads to poor academic performance.

This study was conducted as part of the Taiwan missionaries in the Marshall Islands. One of the limitations of this study is the number of samples collected since the samples from the children were collected only once. Therefore, the sensitivity of positive detection and prevalence of pinworm infection in children may be underestimated that warrants further studies by increasing the number of samples in the future.

## 5. Conclusions

The overall prevalence of pinworm infection is not comparatively high among school-aged children in the Marshall Islands. Although the risk factor is not associated with personal hygiene, it is still important to continuously examine and give personal hygiene education to the community. In addition, the children should be educated and health workers should be trained on how to perform and screen children with enterobiasis symptom-like infection. Additionally, regular deworming of vulnerable children by the Ministry of Health is urgently needed.

## Figures and Tables

**Table 1:** Demographic analysis of family background of *Enterobius vermicularis* infection among primary schoolchildren in the Marshall Islands.

Variables		Total (*N* = 346)	Positive case	Infection rate (%)	*p* value
Gender					
Female		167	22	13.17	0.56
Male		179	20	11.17	

Age (years)					
≤8		143	17	11.89	0.90
>8		203	25	12.32	

Residence					
Urban	Yes	118	7	5.93	0.01^*∗*^
	No	228	35	15.35	

Parent's occupation status					
Father	Yes	268	33	12.31	0.65
	No	55	8	14.55	
Mother	Yes	103	8	7.77	0.10
	No	236	33	13.98	

Siblings					
Elder brother	Yes	251	31	12.35	0.68
	No	93	10	10.75	
Elder sister	Yes	232	32	13.79	0.13
	No	111	9	8.11	
Younger brother	Yes	212	27	12.74	0.57
	No	131	14	10.69	
Younger sister	Yes	183	23	12.57	0.71
	No	160	18	11.25	

^*∗*^Chi-square test.

**Table 2:** Logistic regression analysis of personal hygiene associated with *Enterobius vermicularis* infection among primary schoolchildren in the Marshall Islands.

Variables	EV		
	OR	95% CI	*p* value
Washing hands before eating			
Infrequent	1.00	0.19–2.02	0.43
Frequent	0.62		

Washing hands after using toilet facilities			
Infrequent	1.00	0.32–3.15	0.99
Frequent	1.01		

Finger sucking			
No	1.00	0.69-5.74	0.21
Yes	1.99		

Keeping fingernails short			
No	1.00	0.45–2.45	0.92
Yes	1.05		

Way of bathing			
Showering	1.00	0.54–3.84	0.46
Bathing in a tub	1.44		

Taking a bath after getting up			
No	1.00	0.58–6.69	0.28
Yes	1.96		

Taking a bath every day			
No	1.00	0.13–5.57	0.87
Yes	0.86		

Bathing with the help of family members			
No	1.00	0.48–2.28	0.92
Yes	1.04		

Cleaning house every day			
No	1.00	0.09–2.04	0.29
Yes	0.43		

Type of bed			
Matting	1.00	0.29–1.37	0.24
Wood or spring mattress	0.63		

Changing bedding less than two weeks			
No	1.00	0.04–1.48	0.13
Yes	0.25		

Sharing bedroom with family members			
No	1.00	0.38–2.45	0.94
Yes	0.96		

Sharing bed with family members			
No	1.00	0.76–5.52	0.15
Yes	2.05		

**Table 3:** Logistic regression analysis of risk factors associated with *Enterobius vermicularis* infection among primary schoolchildren in the Marshall Islands.

Variables	EV		
	OR	95% CI	*p* value
Gender			
Female	1.00	0.42–1.87	0.76
Male	0.89		

Age (years)			
≤8	1.00	0.41–2.04	0.82
>8	0.91		

Urban			
No	1.00	0.13–0.93	0.03^*∗*^
Yes	0.35		

Father's occupation			
No	1.00	0.41–2.76	0.91
Yes	1.06		

Mother's occupation			
No	1.00	0.29–1.74	0.45
Yes	0.71		

Having elder brother			
No	1.00	0.38–2.05	0.78
Yes	0.89		

Having elder sister			
No	1.00	0.54–3.03	0.58
Yes	1.27		

Having younger brother			
No	1.00	0.50–2.33	0.85
Yes	1.08		

Having younger sister			
No	1.00	0.44–2.45	0.86
Yes	0.94		

^*∗*^Logistic regression.

## Data Availability

The raw data used to support the findings of this study are available from the corresponding author upon request.

## Abbreviations

PSC: Primary schoolchildren
RMI: Republic of Marshall Islands
SAS: Statistical Analysis System
OR: Odds ratio
CI: Confidence interval.
