# Correction to “Epidemiologic Study of *Enterobius vermicularis* Infection among Schoolchildren in the Republic of Marshall Islands”

**DOI:** 10.1155/jotm/9868907

**Published:** 2025-07-09

C.-K. Fan, P. Sonko, Y.-L. Lee, et al., “Epidemiologic Study of *Enterobius vermicularis* Infection among Schoolchildren in the Republic of Marshall Islands,” *Journal of Tropical Medicine* 2021 (2021): 6273954, https://doi.org/10.1155/2021/6273954.

In the article titled “Epidemiologic Study of *Enterobius vermicularis* Infection among Schoolchildren in the Republic of Marshall Islands,” there was an error in the introduction section. This error is shown below:

“[Enterobiasis is considered the most prevalent helminth infection, with an estimated 1000 million cases worldwide [5]].”

Should read.

“[Enterobiasis is considered the most prevalent helminth infection, with an estimated 100 million cases worldwide [5]].”

